# Sperm-carried IGF2 downregulated the expression of mitogens produced by Sertoli cells: A paracrine mechanism for regulating spermatogenesis?

**DOI:** 10.3389/fendo.2022.1010796

**Published:** 2022-11-29

**Authors:** Rossella Cannarella, Francesca Mancuso, Iva Arato, Cinzia Lilli, Catia Bellucci, Marco Gargaro, Roberto Curto, Maria C. Aglietti, Sandro La Vignera, Rosita A. Condorelli, Giovani Luca, Aldo E. Calogero

**Affiliations:** ^1^ Department of Clinical and Experimental Medicine, University of Catania, Catania, Italy; ^2^ Glickman Urological & Kidney Institute, Cleveland Clinic Foundation, Cleveland, OH, United States; ^3^ Department of Medicine and Surgery, University of Perugia, Perugia, Italy

**Keywords:** IGF2, spermatogenesis, Sertoli cell, spermatozoa, *GDNF*, *SCF*, *FGF2*

## Abstract

**Introduction:**

Insulin-like growth factor 2 (IGF2) mRNA has been found in human and mouse spermatozoa. It is currently unknown whether the IGF2 protein is expressed in human spermatozoa and, if so, its possible role in the cross-talk between germ and Sertoli cells (SCs) during spermatogenesis.

**Methods:**

To accomplish this, we analyzed sperm samples from four consecutive Caucasian men. Furthermore, to understand its role during the spermatogenetic process, porcine SCs were incubated with increasing concentrations (0.33, 3.33, and 10 ng/mL) of recombinant human IGF2 (rhIGF2) for 48 hours. Subsequently, the experiments were repeated by pre-incubating SCs with the non-competitive insulin-like growth factor 1 receptor (IGF1R) inhibitor NVP-AEW541. The following outcomes were evaluated: 1) Gene expression of the *glial cell-line derived neurotrophic factor* (*GDNF*), *fibroblast growth factor 2* (*FGF2*), and *stem cell factor* (*SCF*) mitogens; 2) gene and protein expression of follicle-stimulating hormone receptor (FSHR), anti-Müllerian hormone (AMH), and inhibin B; 3) SC proliferation.

**Results:**

We found that the IGF2 protein was present in each of the sperm samples. IGF2 appeared as a cytoplasmic protein localized in the equatorial and post-acrosomal segment and with a varying degree of expression in each cell. In SCs, IGF2 significantly downregulated *GDNF* gene expression in a concentration-dependent manner. *FGF2* and *SCF* were downregulated only by the highest concentration of IGF2. Similarly, IGF2 downregulated the FSHR gene and FSHR, AMH, and inhibin B protein expression. Finally, IGF2 significantly suppressed the SC proliferation rate. All these findings were reversed by pre-incubation with NVP-AEW541, suggesting an effect mediated by the interaction of IGF2 with the IGFR.

**Conclusion:**

In conclusion, sperm IGF2 seems to downregulate the expression of mitogens, which are known to be physiologically released by the SCs to promote gonocyte proliferation and spermatogonial fate adoption. These findings suggest the presence of paracrine regulatory mechanisms acting on the seminiferous epithelium during spermatogenesis, by which germ cells can influence the amount of mitogens released by the SCs, their sensitivity to FSH, and their rate of proliferation.

## 1 Introduction

Infertility is defined as failure to achieve pregnancy after 12-24 months of unprotected sexual intercourse (1). It affects about 15% of couples of reproductive age (2), with the male factor alone or in combination with the female one contributing to about half of the couple’s infertility cases (3).

One of the most important tests to evaluate the fertility potential of the male partner is the semen analysis, which allows for assessing the conventional sperm parameters, such as sperm concentration, total sperm count, motility, and morphology (4). However, pregnancy may not be achieved in a healthy woman, even in the case of normal conventional sperm parameters. This is in line with the results of assisted reproductive techniques (ARTs) (5). Therefore, other, still poorly understood, mechanisms can affect male fertility. Accordingly, several studies have focused their attention on sperm bio-functional parameters, such as sperm DNA/chromatin integrity, mitochondrial function, etc. (6, 7). However, the assessment of these parameters often fails to identify the cause of male infertility (5).

The causes of oligozoospermia are not defined in up to 75% of cases; therefore, it remains idiopathic (8). Furthermore, according to a meta-regression analysis, sperm concentration and the total sperm count have substantially decreased in the last 40 years, although the causes are unknown (9). This highlights the need to clarify the etiology of male infertility. Therefore, epigenetic, transcriptomic, and proteomic studies are underway to evaluate the molecular etiology of male infertility (10, 11).

The human spermatozoon is a highly differentiated cell characterized by small size, lack of most typical organelles, minimal cytoplasm, compact nucleus, and high motility. It was considered a mere transporter of the paternal genome into the oocyte for several years. However, this cell carries thousands of RNAs and proteins that play a role in embryogenesis (5, 11). Accordingly, the removal of about 90% of sperm RNA resulted in a significant decrease in blastocyst formation rate after intracytoplasmic sperm injection (ICSI), in mice (12). These RNAs derive from non-methylated or hypomethylated genes (5), which the transcriptional apparatus can access. Interestingly, many genes are differentially methylated in infertile patients compared to fertile men (13). Particularly the most investigated pair of differentially methylated genes is *H19*/*IGF2* (14).

*H19* is expressed by the maternal allele and encodes for a long non-coding RNA (lncRNA) that downregulates the *IGF1R* expression and negatively modulates human placental trophoblast cell proliferation (15). On the other hand, the *IGF2* gene is expressed by the paternal allele and encodes for the homonymous protein, which interacts with insulin-like growth factor receptor (IGF1R) and insulin receptor (INSR) (16, 17). Their transcription is regulated by the *H19* differently mediated region (DMR), which is non-methylated in the maternal allele and methylated in the paternal allele. The methylation promotes the *IGF2* expression and inhibits that of *H19* (5, 18).

According to another study, higher levels of IGF2 mRNA are present in spermatozoa from normozoospermic men than in oligozoospermic patients (19) but there is no evidence of IGF2 protein expression. In addition, a lower *H19* DMR methylation rate in spermatozoa has been reported in male partners of women with idiopathic recurrent pregnancy loss (RPL) compared to male partners of control couples (20). However, a cause-and-effect relationship between these associations has not been demonstrated.

IGF2 is a protein belonging to the insulin-like growth system and binds to IGF1R, as do IGF1 and insulin (21). Evidence supports that the IGF system could affect testicular differentiation, Sertoli cell (SC) and germ cell (GC) proliferation, and GC differentiation in mice. Furthermore, IGF1R appears to mediate the effects of FSH *via* the PI3K/AKT pathway (22). Moreover, covalent inhibition of IGFR1 prevents FSH-induced AKT phosphorylation and GC proliferation in female mouse gonads (23). Although IGF2 mRNA is known to be expressed in human spermatozoa (19), no studies have explored whether the IGF2 protein is also present in these cells.

Numerous data indicate that GCs are sources and targets of signaling molecules, such as growth factors, cytokines, and peptides (24, 25). These observations, which have been partially confirmed by *in-vivo* models using gene knockout or overexpression (24), suggest that some key steps in spermatogenesis are controlled by regulatory factors. Some mitogens that promote self-renewal and differentiation of spermatogonial stem cells (SSCs) are produced by SCs, such as the glial cell-line derived neurotrophic factor (GDNF) (26, 27), fibroblast growth factor 2 (FGF2) (28, 29), and stem cell factor (SCF) (30).

GDNF synthesis depends on follicle-stimulating hormone (FSH) levels (27). It works by binding to its receptors, GFRA1, and c-RET, on the plasma membrane of spermatogonia (26, 31). This activates the intracellular signaling pathway PI3K/AKT and SFK, affecting transcription factors, such as *B cell CLL/lymphoma 6 member B* (*BCL6B*), *ETS variant 5* (*ERM*; also known as *ETV5*), *DNA*-*binding protein 4* (*ID4*), *LIM homeobox 1* (*LHX1*), *BRACHYURY* (T*)*, and *POU class 3 homeobox 1* (*POU3F1*) (32). Through these factors, *GDNF* promotes SSC self-renewal and/or inhibits their differentiation. Furthermore, GDNF promotes the proliferation of SSCs and of type A_paired_ and type A_aligned_ spermatogonia (33–36) and induces the migration of SSC by acting as a chemoattractant (37–39), according to an *in-vitro* study. Disruption of *GDNF* signaling in the testis leads to depletion of SSCs and GC-free seminiferous tubules (40–43).

FGF2 is produced by SCs, peritubular, Leydig cells, and GCs (28, 29). In SCs, as for GDNF, FGF2 production relies on FSH levels (28). FGF2 induces MAP2K1 phosphorylation that activates the MAP2K1 pathway. The latter upregulates the expression of *Etv5*, *Bcl6b*, and *Lhx1*, all of which play crucial roles in SSCs self-renewal (32, 44, 45).

SCF with its receptor, c-KIT, plays an important role in spermatogenesis. Without a functioning *SCF*/c-KIT pathway, A_s-pr-al_ spermatogonia can proliferate as A_al_ spermatogonial clones, but they cannot differentiate into A1 spermatogonia (46, 47).

According to the mechanisms described above, defects in the expression and/or action of these growth factors can be a possible cause of male infertility. As previously mentioned, IGF1R appears to mediate the effects of FSH (48), which in turn influences the secretion of GDNF, FGF2, and SCF in SCs. This could suggest that IGF2, interacting with IGF1R, may interfere with the FSH pathway and mitogen production in SCs. However, this topic has not been explored so far. Therefore, this study aimed to evaluate whether IGF2 is expressed in human spermatozoa and, if so, to evaluate its effects on the expression of *GDNF*, *FGF2*, and *SCF* expression in SCs. To achieve this, we examined the mRNA levels of GDNF, FGF2, and SCF in porcine SCs after incubation with increasing IGF2 concentrations. *Follicle-stimulating hormone receptor* (*FSHR*) gene expression, *anti-Müllerian hormone* (*AMH*) and *inhibin B* gene expression and protein secretion, and SC proliferation were also analyzed as secondary outcomes.

## 2 Experimental section

### 2.1 Experimental design

The first part of the experimental design was carried in humans. Four men were enrolled and their semen sample was collected to understand if the IGF2 mRNA and protein are expressed in human sperm. Hence, we designed an *in vitro* study on porcine SCs to understand its influence on SC function and regulation of spermatogenesis.

Porcine SCs were isolated from pre-pubertal neonatal (7-15 days of age) Large White pigs using established methods (49, 50). These cells were incubated with increasing concentrations (0.33 ng/mL, 3.33 ng/mL, 10 ng/mL) of recombinant human IGF2 (rhIGF2) for 48 hours. Subsequently, all steps were repeated by pretreating the SCs with the non-competitive IGF1R inhibitor NVP-AEW541 at a concentration of 1 µM, which was added 1 hour before rhIGF2. The following outcomes were assessed: 1) Expression of the mitogenic genes *GDNF*, *FGF2*, and *SCF* by real-time PCR (RT-PCR); 2) Gene (by RT-PCR) and protein [by both Western Blot (WB) and Immunofluorescence (IM)] expression of FSHR; 3) SC proliferation rate by flow cytometry analysis.

### 2.2 Experiments performed in human spermatozoa

#### 2.2.1 Patients

Semen samples from four Caucasian patients attending the Division of Endocrinology, Metabolic Diseases and Nutrition, Department of Clinical and Experimental Medicine, University of Catania, for semen analysis were consecutively recruited. As this was an exploratory study aimed at assessing whether IGF2 mRNA was present in human spermatozoa, no exclusion criteria were adopted. However, each patient’s medical history was carefully collected.

#### 2.2.2 Sperm analysis and selection by “swim-up”

The semen samples were collected by masturbation in a sterile container after 3-4 days of sexual abstinence. Each sample was evaluated for conventional sperm parameters according to World Health Organization (WHO) criteria (4). After liquefaction, 1 ml aliquots of the semen samples were washed using the K-SIMS-50 Sperm Medium (Sydney IVF. William A. Cook, Queensland, Australia) and centrifuged for 15 minutes at 500 g. After removing the supernatant, 1 ml of culture medium was placed on the sperm pellet of each aliquot. The tubes were incubated at 37° C for 30-60 min and 5% CO_2_ for 45 minutes, tilted at an angle of approximately 45°. At the end of the incubation, 1 ml of the supernatant was gently removed from each tube (51). The spermatozoa were then washed in 1 x phosphate-buffered saline (PBS) and used for subsequent experiments.

#### 2.2.3 RT-PCR analysis

Total RNA was extracted and quantified by reading the optical density at 260 nm. Specifically, 2.5 μg of total RNA was reverse transcribed (RT, Thermo Scientific, Waltham, MA, USA) to a final volume of 20 μl. qPCR was performed using 50 ng of the cDNA prepared by RT and an SYBR Green Master Mix (Stratagene, Amsterdam, The Netherlands-Agilent Technology). This was performed in an Mx3000P termal cycler (Stratagene) using FAM for detection and ROX as the reference dye. The mRNA level of each sample was normalized against the β-actin mRNA and expressed as a fold change from the level in untreated control cells. The following primers were used for RT-PCR analysis: IGF2 forward primer 5’-CCCGTGGGCAAGTTCTTCC-3’, reverse primer 5’-CGCTGGGTGGACTGCTTC-3’.

#### 2.2.4 Western blot analysis

Sperm lysates were collected in radioimmunoprecipitation assay (RIPA) lysis buffer (Santa Cruz Biotechnology Inc., Santa Cruz, CA, USA). The mixture was centrifuged at 1000 x *g* (Eppendorf, Hamburg, Germany) for 10 min, the supernatant was collected and total protein content was measured by the Bradford method (52). Sample aliquots were stored at -20°C for Western Blot (WB) analysis. Cell extracts were separated by 4%-12% SDS-PAGE and equal amounts of protein (70 μg protein/lane) were run and blotted on nitrocellulose membranes (BioRad, Hercules, CA; USA). The proteins were separated and transferred to nitrocellulose membranes using an iBlotTM 2 Dry Blotting System (Thermo Fisher, Waltham, MA, USA). After blocking the membranes with 5% dry milk in 10 mM Tris–HCl (pH 8), 0.5 M NaCl, and 1% Tween-20 (TBS), the membranes were incubated with a mouse anti-IGF2 primary antibody (MA5-17096, clone 8H1, dilution factor 1:500), (Invitrogen, Carlsbad, CA, USA). After being washed with TBS containing 1% Tween-20, the blots were incubated with anti-mouse peroxidase-conjugated secondary antibodies (HRP) (1:5000, Santa Cruz Biotechnology Inc., Dallas, TX, USA) and developed using enhanced chemiluminescence (ECL; Bio-Rad, Hercules, CA, USA), according to the manufacturer’s instructions. Porcine SCs were used as positive controls since SCs are known to express Igf2 (53).

#### 2.2.5 Immunofluorescence analysis

The spermatozoa were spread on microscope slides, air dried at room temperature (RT), and fixed in absolute methanol for 10 min at -20°C. The fixed cells were then permeabilized (0.2% Triton x-100 in PBS, Sigma Aldrich, St. Louis, MO, USA) for 10 min at room temperature and blocked with 0.5% BSA in PBS (Sigma Aldrich, St. Louis, MO, USA) for 1 hour at exposure to mouse anti-IGF2 antibody, (MA5-17096, clone 8H1, dilution factor 1:200) (Invitrogen, Carlsbad, CA, USA) at 4°C, overnight. Subsequently, the cells were incubated with a donkey anti-mouse IgG (H&L) DyLight® 488-conjugated secondary antibody (1:500, Thermo Fisher Scientific, Waltham, MA, USA), treated with Rnase (10 mg/ml, Sigma Aldrich, St. Louis, MO, USA), and counterstained for 1 min with 4’,6-diamidino2-phenylindole (DAPI, Sigma Aldrich, St. Louis, MO, USA). Cells were mounted with Prolong® Gold antifade reagent (Molecular Probes, Eugene, OR, USA). IGF2-positive cells were visualized under a BX-41 microscope (Olympus, Tokyo, Japan) equipped with a fluorescence camera (F-viewer, Olympus, Tokyo, Japan); images were processed with the Cell F imaging software (Olympus, Tokyo, Japan).

### 2.3 Experiments performed in porcine Sertoli cells

#### 2.3.1 Sertoli cell isolation, culture, characterization, and function

Testes were removed from Danish Duroc neonatal (7-15 days) Large White pigs. The fibrous capsule was removed. Then, the testes were finely minced and digested twice enzymatically, with a mixed solution of trypsin and deoxyribonuclease I (DNase I) (Sigma-Aldrich, St. Louis, MO, USA) in Hank’s balanced salt solution (HBSS; Merck KGaA, Darmstadt, Germany) and collagenase P (Roche Diagnostic S.p.A., Monza, Italy). The tissue pellet was centrifuged, passed through a stainless-steel mesh with 500 μm pores, and resuspended in glycine to eliminate residual Leydig and peritubular cells (54). The pellet was then collected and kept in Ham’s F12 medium (Euroclone, Milan, Italy) and added with 0.166 nmol L^-1^ of retinoic acid (Sigma-Aldrich Darmstadt, St. Louis, MO, USA) and 5 mL per 500 mL insulin-transferrin-selenium (ITS, Becton Dickinson cat. no. 354352; Franklin Lakes, NJ, USA) in 95% air/5% CO_2_ at 37°C. Cells were cultured for 3 days. Therefore, the purity and functional competence of the SC monolayers were evaluated according to previously established methods (50). In particular, purity was assessed by immunofluorescence analysis of specific markers for both SC (AMH positivity and vimentin positivity >95 ± 2%) and non-SC cells (3β-HSD for Leydig cells and ASMA for peritubular cells <5 ± 2%), (data not shown).

Functional competence was assessed by ELISA assay for two specific SC markers (AMH and inhibin B as presented in the Discussion).

#### 2.3.2 Culture and treatment

When SC monolayers were confluent (after 3 days of culture), they underwent the following treatments: (1) 0.33 ng/ml of rhIGF2 (Cayman Chemical, Ann Arbor, MI, USA) for 48 hours; (2) 3.33 ng/ml of rhIGF2 for 48 hours; (3) 10 ng/ml of rhIGF2 for 48 hours; (4) 1 µM of NVP-AE541 for 1 hour and then incubated with 0.33 ng/ml of rhIGF2 for 48 hours; (5) 1 µM of NVP-AE541 for 1 hour and then incubated with 3.33 ng/ml of rhIGF2 for 48 hours; (6) 1 µM of NVP-AE541 for 1 hour and then incubated with 10 ng/ml of rhIGF2 for 48 hours. The concentrations of rhIGF2 (0.33, 3.33, and 10 ng/mL) were chosen according to the curve of rhIGF2 bioactivity. In particular, the concentration of 3.33 ng/mL was used since it corresponded to the ED_50_. We also used the concentration of 10^1^ ng/mL as it is the one at which IGF2 reaches its highest bioactivity and the concentration of 0.33 ng/ml which corresponds to the concentration at which IGF2 starts to exert its bioactivity. Finally, the time and the concentration of NVP-AE541 were used according to previous experiments (48).

#### 2.3.3 RT-PCR analysis

Total RNA was extracted and quantified, following the same procedure described in section 2.2.3.

The following primers were used for RT-PCR analysis: AMH, forward primer 5’-GCGAACTTAGCGTGGACCTG-3’, reverse primer 5’-CTTGGCAGTTGTTGGCTTGATATG-3’; Inhibin B, forward primer 5’-TGGCTGGAGTGACTGGAT-3’, reverse primer 5’-CCGTGTGGAAGGATGAGG-3’; FSHR forward primer 5’-TTTCACAGTCGCCCTCTTTCCC-3’, reverse primer 5’-TGAGTATAGCAGCCACAGATGACC-3’; GDNF forward primer 5’-TCAAGCCACCATCAGAAGA -3’ reverse primer 5’-TAGCCCAAACCCAAGTCA-3’; FGF2 forward primer 5’-CCTCACATCAAACTACAACTTCA-3’ reverse primer 5’-TCTTCCATCTTCCTTCATAGCA-3’; SCF forward primer 5’-GAATGACAGCAGTAGCAGTAAT-3’ reverse primer 5’-TTCTTCCAGTATAAGGCTCCAA-3’; actin, forward primer 5’-ATGGTGGGTATGGGTCAGAA-3’, reverse primer 5’-CTTCTCCATGTCGTCCAGT-3’.

#### 2.3.4 Western blot analysis

At the end of the incubation period, total cell lysates were collected in radioimmunoprecipitation assay (RIPA) lysis buffer (Santa Cruz Biotechnology Inc., Santa Cruz, CA, USA). The protocol used is the same described in section 2.2.4. The NBP2-36489 FSHR antibody (6E8.2F5) was used (dilution factor 1:300) (Novus Biologicals, Centennial, CO, USA).

#### 2.3.5 Immunofluorescence analysis

The procedure described in section 2.2.5 was performed on SCs to define the expression of FSHR. After cell fixation, SCs were blocked with 0.5% BSA in PBS (Sigma Aldrich, St. Louis, MO, USA) for 1 hour upon exposure to rabbit anti-FSHR polyclonal antibody, dilution 1:300 (Novus Biologicals, Saint Charles, MO, USA), at 4°C. For FACS analysis, porcine SC monolayers were harvested and centrifuged (400 g for 5 min) to form a cell pellet of approximately 10^6^ cells. The cells were fixed in 4% PFA-PBS for 30 min and then treated with 0.1% Triton X-100 in FACS buffer for 10 min. After centrifugation (400 g for 5 min), the cells were blocked with 5% BSA in FACS buffer at RT for 1 h before incubation with primary antibody (FSHR 1 µl antibody per 5x10^5^ to 1.0 x10^6^ cells, or buffer alone) at RT for 1 h. Finally, SCs were incubated with the Alexa 488-conjugated donkey anti-rabbit secondary antibody (1:500) for 30 minutes. Data acquisition was performed on 20,000 events per tube based on a total (gated alive cells) count of forward and side light scatter at approximately 200-300 events per second on a BD FAC Sort flow cytometer (BD Biosciences), analyzed using FACS Diva software (BD Biosciences, Franklin Lakes, NJ, USA) and gated on appropriate controls in different cell populations.

#### 2.3.6 AMH and inhibin B secretion assay

Aliquots of the culture media of treated and untreated SCs were stored at −20°C for the assessment of AMH (AMH Gen IIELISA, Beckman Coulter; intra-assay CV = 3.89%; inter-assay CV = 5.77%) and inhibin B (Inhibin B Gen II ELISA, Beckman Coulter, Webster, TX, USA; intra-assay CV =2.81%; inter-assay CV= 4.33%) secretion as previously described (50).

#### 2.3.7 Flow cytometry analysis

After reaching 50-60% confluence, cells were treated with 0.1 μg/mL colcemid (Sigma-Aldrich, St. Louis, MO, USA) for 3 hours in the incubator (55) and washed with phosphate buffer (PBS). For the cell proliferation assay, SCs were incubated with 1 μM 5(6)-carboxyfluorescein diacetate N-succinimidyl ester (CFSE, Sigma-Aldrich, St. Louis, MO, USA) in PBS for 8 min and washed with HBSS medium three times. Subsequently, the CFSE-labeled SCs were cultured at 37°C, 5% CO_2_ incubator for 48 hours following the established protocol of stimulations (AB, rhIGF2 (0,33 ng/ml), rhIGF2 (0,33 ng/ml) + AB, rhIGF2 (3,33 ng/ml), rhIGF2 (0,33 ng/ml) + AB, rhIGF2 (10 ng/ml), rhIGF2 (10 ng/ml) + AB). At the end of the stimulation protocol, the cells were washed with PBS, harvested by trypsinization, and then counted using an Automated Cell Counter (Invitrogen, Carlsbad, CA, USA) before to flow cytometer analysis (56). Data acquisition was performed on 20,000 events per tube based on a total (gated alive cells) count of forward and side light scatter at approximately 200-300 events per sec on a BD FAC Sort flow cytometer (BD Biosciences), analyzed using FACS Diva software (BD Biosciences, Franklin Lakes, NJ, USA), and gated on appropriate controls in different cell populations.

#### 2.3.8 Ethics statement

This study was conducted in strict compliance with the Guide for the Care and Use of Laboratory Animals of the National Institutes of Health and the University of Perugia Animal Care. The protocol was approved by the internal Institutional Ethics Committee (Ministry of Health authorization n. 971/2015-PR, 9/14/2015).

#### 2.3.9 Statistical analysis

Results are shown as mean ± SD of three independent experiments, each one performed in triplicate. Data were analyzed for statistical significance by one-way ANOVA, followed by a Turkey *post hoc* test using SPSS 9.0 for Windows (SPSS Inc., Chicago IL, USA). Significance was accepted for a *p-*value lower than 0.05.

## 3 Results

### 3.1 Expression of the IGF2 mRNA and protein in human spermatozoa

The conventional sperm parameters of the four consecutive Caucasian patients *(27.2 ± 3.6 years old)* enrolled are shown in **Table 1**. Patient 1 was the partner of a woman with idiopathic RPL. Patients 2 and 3 were not interested in having a child. Patient 4 and his partner sought pregnancy for 11 months before being enrolled in this study.

**Table 1:** Conventional sperm parameters of the semen samples of the men enrolled in this study.

ID	Sperm concentration (million/ml)	Total sperm count (million/ejaculate)	Progressive motility (%)	Total motility (%)	Normal forms (%)	Leukocyte concentration (million/ml)
1	65	292.5	32	61	14	0.65
2	67	167.5	32	50	5	1.34
3	2	6	16	60	7	0.12
4	110	385	25	75	10	0
L.L.	>15	>39	>32	>40	>4	<1

L.L., lower limit according to the WHO criteria (WHO 5^th^ manual, 2010).

**Figure 1:**
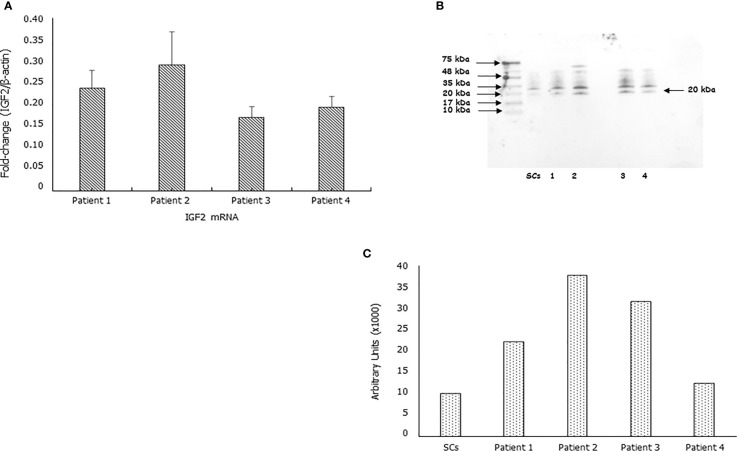
IGF2 mRNA and protein are expressed in human spermatozoa. RT-PCR analysis **(A)**, immunoblots **(B)**, and densitometric analysis **(C)** of IGF2 in human spermatozoa. MA5-17096, clone 8H1, detects IGF2 which has a predicted molecular weight of approximately 20.1 kDa. The observed molecular weight of the protein may vary from the mentioned predicted molecular weight due to post-translational modifications, post translation cleavages, relative charges, and other experimental factors, as shown by the presence of other bands at the highest molecular weight (in particular at 35 KDa). Data represent the mean ± SD of three independent experiments, each performed in triplicate. Porcine Sertoli cells (SCs) were used as a positive control. Magnification 100x.

IGF2 mRNA was found expressed in each sample. After normalizing for the β-actine, the fold-change of the IGF2 mRNA was 0.24 ± 0.04, 0.29 ± 0.08, 0.17 ± 0.02, 0.19 ± 0.02in Patient 1, 2, 3 and 4, respectively (**Figure 1A**). Furthermore, the IGF2 protein was found by Western blot and immunofluorescence analysis in all samples (**Figures 1B, C** **)**.

**Figure 2:**
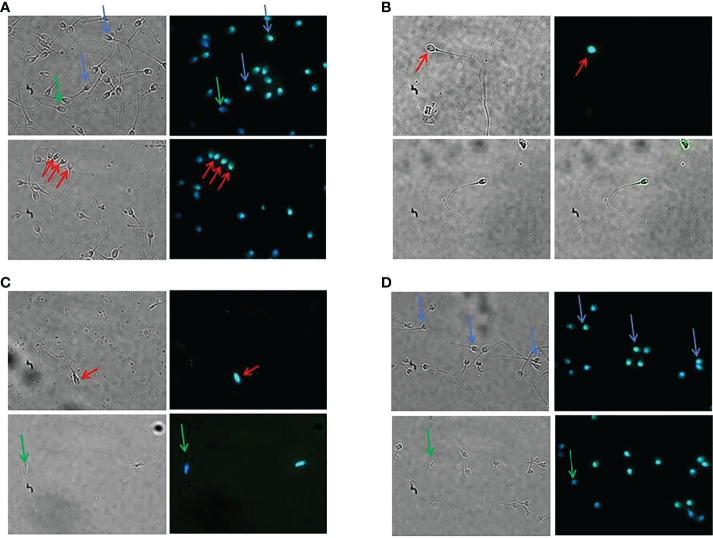
Immunolocalization of the IGF2 protein in human spermatozoa. Immunofluorescence analysis of spermatozoa of patients 1 **(A)**, 2 **(B)**, 3 **(C)**, and 4 **(D)**. The protein showed a cytoplasmic, equatorial, and post-acrosomal localization (red arrows). Green arrows indicate spermatozoa lacking IGF2 protein. Blue arrows indicate a different degree of immunofluorescence in different spermatozoa of the same patient. Magnification 100x.

IGF2 appeared as a cytoplasmic protein with equatorial and post-acrosomal localization. Interestingly, the protein was not visible in all spermatozoa being evident only in some of them (albeit at various levels of expression) and completely absent in others (**Figure 2**).

### 3.2 Effects of IGF2 on mitogens expression

Once the presence of IGF2 as a cytoplasmic protein with equatorial and post-acrosomal localization was clarified, the second aim of the study was to explore the effects of this protein on SCs, to understand consequently how it can influence the regulation of spermatogenesis.

Therefore, in this section, we report the effects of IGF2 on the expression of mitogens secreted by SCs, with or without pre-treatment with the IGF1R inhibitor NVP-AEW541.

**Figure 3:**
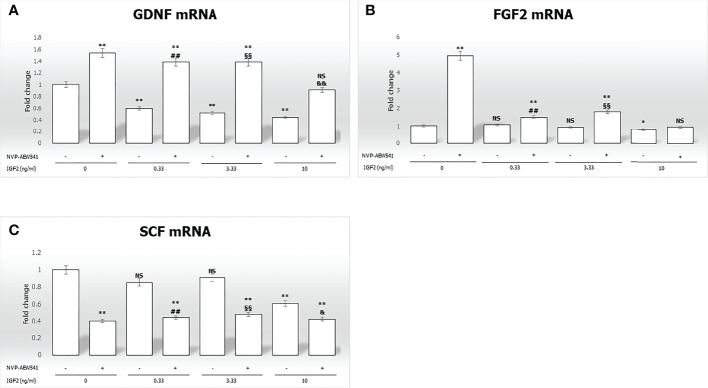
Gene expression of the mitogens Glial cell-line derived neurotrophic factor *(GDNF)*, *Fibroblast growth factor 2 (FGF2)*, and *Stem cell factor (SCF)* in Sertoli cells (SCs). RT-PCR analysis of **(A)** *GDNF*, **(B)** *FGF2*, and **(C)** *SCF*. Data represent the mean ± SD of three independent experiments, each performed in triplicate. SCs were used as a positive control. Non-significant (NS) *vs* SCs, *p < 0.05 and **p < 0.001 *vs* SCs, ##p < 0.001 *vs* 0.33 ng/ml of rhIGF2, §§p < 0.001 *vs* 3.33 ng/ml, &p < 0.05 and &&p < 0.001 *vs* 10 ng/ml of rhIGF2, ns *vs* 10 ng/ml of rhIGF2.

IGF2 treatment significantly downregulated *GDNF* gene expression in a concentration-dependent fashion (-40.3% at 0.33 ng/mL, -48.2% at 3.33 ng/mL, -55.5% at 10 ng/mL, respectively, p<0.01). Gene expression of *FGF2* and *SCF* was downregulated only by IGF2 to the highest concentration used (-23.6% and -39.1%, respectively, p<0.01). The effects of IGF2 on the gene expression of *GDNF*, *FGF2*, and *SCF* were reversed after incubation with NVP-AEW541 (**Figure 3**).

### 3.3 Effects of IGF2 on anti-Müllerian hormone and inhibin B gene expression and secretion

In this section, gene expression and protein secretion of *AMH* and *inhibin B* were evaluated in pre-pubertal porcine SCs following incubation with increasing IGF2 concentrations, with or without pre-treatment with NVP-AEW541.

**Figure 4:**
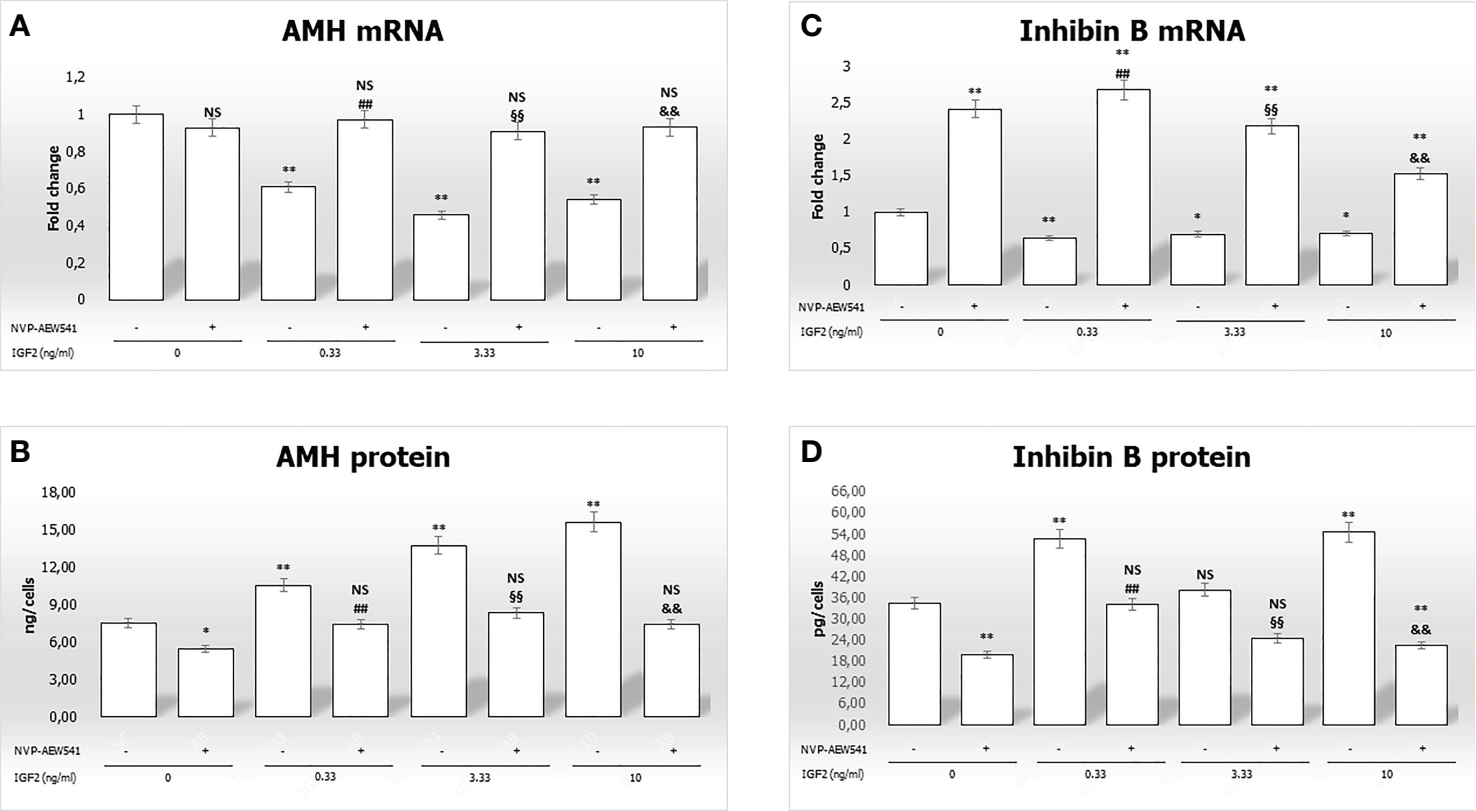
Anti-Müllerian hormone (AMH) and inhibin B gene expression and protein secretion. AMH mRNA and protein levels are shown in panels **(A, B)**, respectively. Inhibin B mRNA and protein levels are shown in panels **(C, D)**, respectively. Data represent the mean ± SD of three independent experiments, each performed in triplicate. Non-significant (NS) vs SCs, *p < 0.05 and **p < 0.001 vs SCs, ##p < 0.001 vs 0.33 ng/ml of rhIGF2, §§p < 0.001 vs 3.33 ng/ml, and &&p < 0.001 vs 10 ng/ml of rhIGF2.

The gene expression of *AMH* and *inhibin B* gene expression was reduced by incubation with IGF2 (**Figures 4A, C** **)**. Differences in AMH mRNA levels with the untreated control were -39.2% at 0.33 ng/mL, -54.4% at 3.33 ng/mL, and -55.9% at 10 ng/mL (p<0.01). Similar effects were observed with inhibin B mRNA levels (-35.5% at 0.33 ng/mL, -30.4% at 3.33 ng/mL, -29.7% at 10 ng/mL, p<0.01). Finally, pre-treatment with NVP-AEW541 and incubation with IGF2 restored the expression of *AMH* and *inhibin B* at each concentration.

**Figure 4B**, shows a significantly higher secretion of AMH after incubation with IGF2 (10.5 ng/cells at 0.33 ng/mL, 13.7 ng/cells at 3.33 ng/mL, and 15,5865 ng/cells at 10 ng/mL) compared to untreated controls (7.5 ng/cells). Similar effects were observed for inhibin B secretion (**Figure 4D**) (52.7 ng/cells at 0.33 ng/mL, 38.2 ng/cells at 3.33 ng/mL, and 54.5 ng/cells at 10 ng/mL vs. 34.4 ng/cells). Finally, pre-treatment with NVP-AEW541 and incubation with IGF2 decreased the secretion of AMH and inhibin B.

### 3.4 IGF2 regulates the sensitivity of Sertoli cells to FSH by modulating the expression of its receptor

In this section, we focused on the regulation of the FSHR gene and protein expression in SCs incubated with increasing concentrations of IGF2, with or without pre-treatment with the IGF1R inhibitor NVP-AEW541.

**Figure 5:**
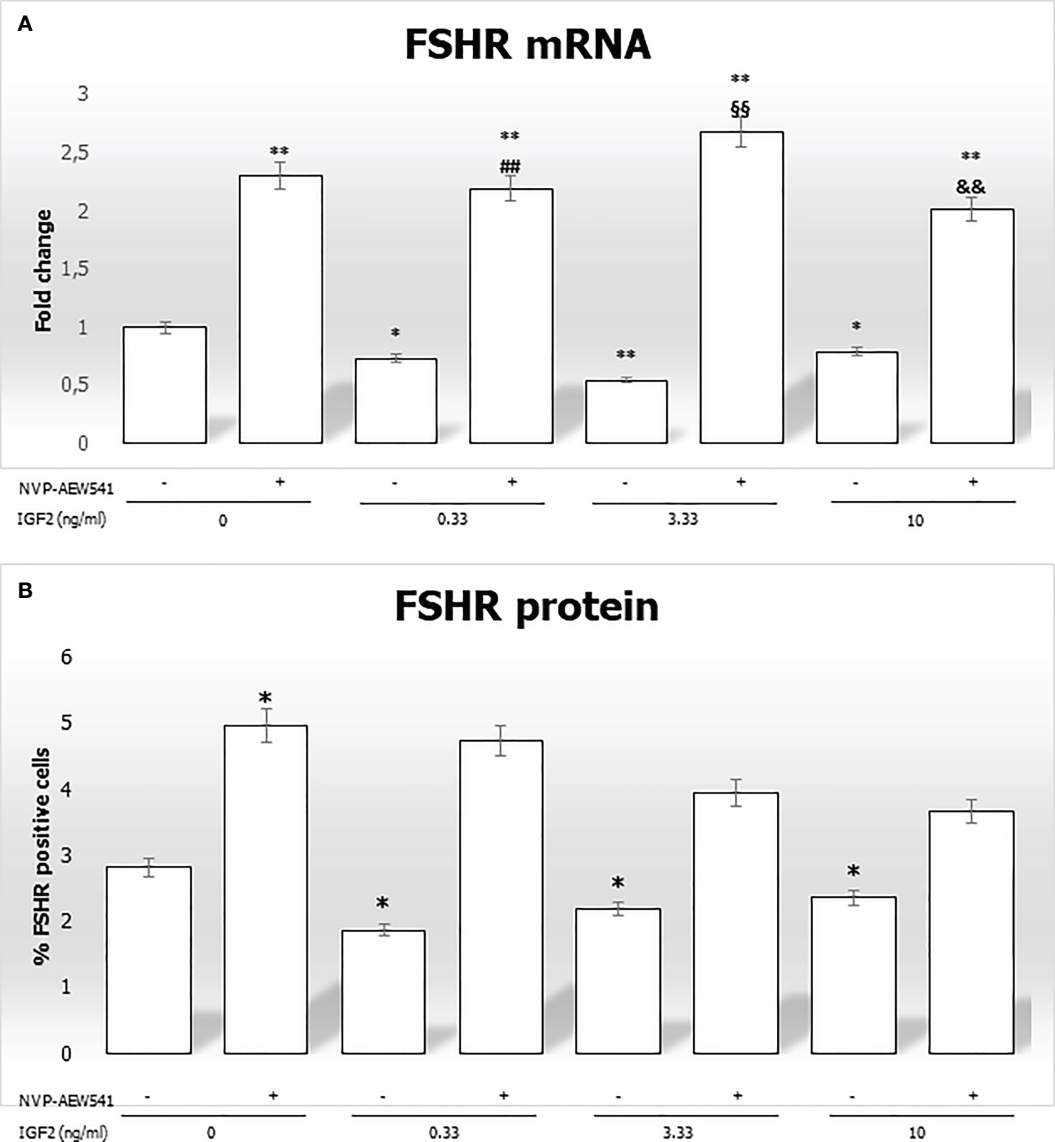
FSHR gene and protein expression in Sertoli cells (SCs). **(A)** RT-PCR analysis of the FSHR gene. Data represent the mean ± SD of three independent Q3 experiments, each performed in triplicate. **(B)** Cytofluorimetric analysis. The bars represent the percentage of FSHR-positive cells without stimulation, after incubation with NVP-AEW541, IGF2, or NVP-AEW541 + IGF2. Compared with the control, the percentage of FSHR-positive cells was significantly reduced after incubation with IGF2 (2.82 ± 0.1% in the untreated controls, 1.87 ± 0.1% at 0.33 ng/mL, 2.19 ± 0.2% at 3.33 ng/mL, 2.36 ± 0.1% at 10 ng/mL; p < 0.01). The suppression was reversed after incubation with NVP- AEW541. Data represent the mean ± SD of three independent experiments, each performed in triplicate. FSHR, follicle-stimulating hormone receptor; IGF2, Insulin-like growth factor 2. *p < 0.05 and **p < 0.001 vs SCs, ##p < 0.001 vs 0.33 ng/ml of rhIGF2, §§p < 0.001 vs 3.33 ng/ml, &&p < 0.001 vs 10 ng/ml of rhIGF2”

**Figure 6:**
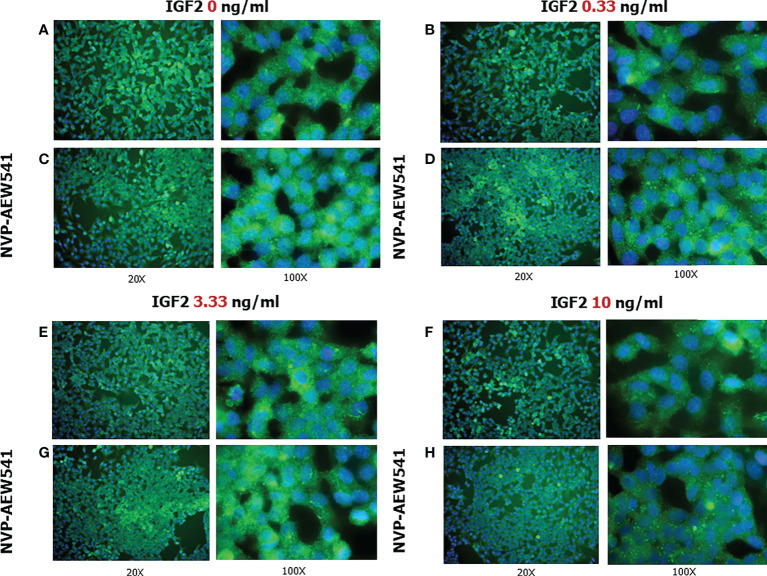
FSHR protein expression in Sertoli cells (SCs). Immunofluorescence analysis of the FSHR protein expression without treatment **(A)** or after incubation with NVP-AEW541 **(B)**, IGF2 0.33 ng/ml **(C)**, NVP-AEW541 + IGF2 0.33 ng/ml **(D)**, IGF2 3.33 ng/ml **(E)**, NVP-AEW541 + IGF2 3.33 ng/ml **(F)**, IGF2 10 ng/ml **(G)**, and NVP-AEW541 + IGF2 10 ng/ml **(H)**. Blu: DAPI (nuclei), Green: dy-light 488. Magnification 20x (left column) and 100x (right column) for each panel. The positive control is shown in Panel **(A)**, upper right and left. The negative control is shown in **Supplementary Figure 1**.

IGF2 incubation downregulated the *FSHR* gene expression (-27.1% at 0.33 ng/mL, -45.4% at 3.33 ng/mL, and -21.1% at 10 ng/mL, p<0.01). This was reversed by pre-incubation with NVP-AEW541 (**Figure 5A**). Compared to untreated control, the FSHR expression is downregulated after the incubation with IGF2, which was reversed after incubation with NVP-AEW541 (**Figure 5**). The same results were observed for the FSHR protein by both cytofluorimetric assay (**Figure 5B**) and IF (**Figure 6**).

### 3.5 Sertoli cell number and proliferation

In this final section, we evaluated the effects of IGF2 on SC proliferation, with or without pre-treatment with the IGF1R inhibitor NVP-AEW541.

**Figure 7:**
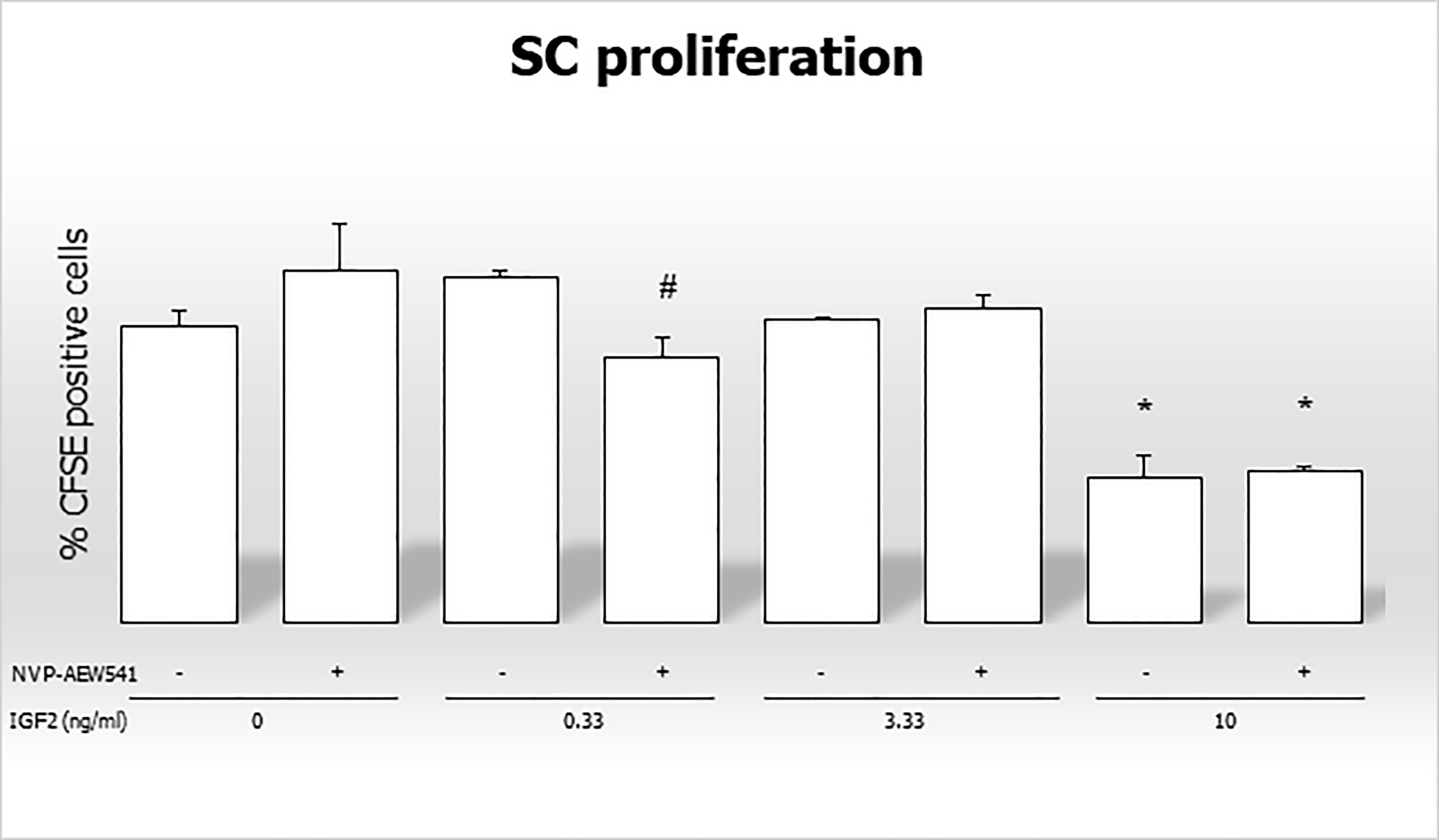
Flow cytometric analysis of Sertoli cells stained with CFSE without stimulation, after incubation with IGF2, NVP-AEW541, and IGF2 + NVP-AEW541. *p < 0.05 *vs* SCs, #p < 0.05 *vs* 0.33 ng/ml of rhIGF2.

The percentage of CFSE positive cells decreased after incubation with 0.33 ng/mL of IGF2 and NVP-AEW541, and with 10 ng/mL of IGF2 and NVP-AEW541 (70,886% in untreated controls; 69,596% in NVP-AEW541 + 0.33 ng/mL of IGF2; 64,326% in 10 ng/mL of IGF2; 64,643 in NVP-AEW541+ 10 ng/mL of IGF2) (**Figure 7**).

## 4 Discussion

### 4.1 Sperm IGF2 and ICSI outcomes

The data from this study showed that mRNA and protein IGF2 are expressed in human spermatozoa, suggesting that the *IGF2* gene is both transcribed and translated. This is more likely to happen in spermatogonia or spermatocytes, during the early phases of spermatogenesis, due to the highly degree of chromatin compactness in spermatozoa (57). However, the possibility that these processes may even partially occur in spermatozoa cannot be completely ruled out as some regions of the sperm genome are accessible to the transcriptional machinery (58). Interestingly, the *IGF2* gene was not ubiquitously expressed in all spermatozoa. Indeed, some spermatozoa had higher IGF2 expression than others, while other cells showed no expression, even in the same patient. Research is needed to better understand whether different amounts of sperm-carried IGF2 could affect ART outcomes.

This hypothesis is based on the effects of IGF2 on oocyte maturation. According to a previous study, incubation with IGF2 ameliorates oocyte mitochondrial function and meiotic maturation, reduces the levels of reactive oxidative species, and promotes a better chromosome alignment in the early embryo in mice, resulting in an improvement in the rate of blastocyst formation following ICSI performed in the oocytes of aged mice (59).

Furthermore, the equatorial and post-acrosomal localization of the IGF2 protein within spermatozoa is compatible with its release into the cytoplasm of oocyte following fertilization. The role of IGF2 in embryo development is well established as it binds its receptors (IGF1R and INSR), induces receptor phosphorylation, and activates the PI3K/Akt signaling pathway. This promotes embryo development and cell proliferation (60–63). Consequently, mouse models with a ~10 kb deletion that includes the *H19* gene, causing *Igf2* gene overexpression, showed a 145% and 123% increase in placental and embryo growth, respectively, compared to wild-type animals (64). These findings strongly suggest that *IGF2* gene expression plays an important role in embryo-placental development and provide the basis for studying the relationship between sperm IGF2 levels and blastocyst formation rate and/or embryo growth velocity. Consistent with this, in our study, the male partner of a woman with RPL showed lower levels of the IGF2 protein. Certainly, this deserves to be confirmed by further studies with a larger sample size specifically aimed at evaluating the association between RPL and low sperm IGF2 levels. Interestingly, some studies have already shown lower *H19* DMR methylation (which results in low *IGF2* expression (5)) in the spermatozoa of male partners of couples with RPL (20, 65). Collectively, these findings suggest the importance of further exploring the relationship between sperm IGF2 and ICSI outcomes.

### 4.2 FSH and IGF2 synergistically regulate gene expression in a PKA-dependent manner

The PI3K signaling pathway regulates transcription, translation, proliferation, and apoptosis (66, 67). This pathway is activated by tyrosine kinases receptors, such as IGF1R, and G-protein-coupled receptors (GPCRs), such as FSHR. Studies have shown that IGF1 (in rodents) (68) and IGF2 (in humans) (69) are produced by GCs and are required for the expression of the FSH-dependent target gene. After IGF2 activation of IGF1R, adapter proteins will be phosphorylated, such as insulin receptor substrate 1 (IRS1), to facilitate the activation of downstream targets (70). One of these is PI3K (71, 72), which activates the AKT kinase that plays a central role in many processes, including regulation of transcription and translation (66). Furthermore, AKT mediates the downstream synergy between FSH and IGF1 by inhibiting the transcriptional repressor forkhead box protein O1 (FOXO1) (66), which represses the expression of several genes in SCs (73–75).

After binding its receptor, FSH activates adenylate cyclase that increases the cAMP intracellular content. The latter leads to the activation of PKA that phosphorylates myosin-phosphatase 1 (MYPT1) (48, 76). This is a protein made up of three subunits; the PP1c, a targeting/regulatory subunit, and a 20kDa subunit of unknown function called M20 (77, 78). Through MYPT1, PKA activates the protein phosphatase 1 β (PP1β) (76), which dephosphorylates the inhibitory Ser/Thr residues on IRS, thereby permitting IGF1R to phosphorylate IRS1 to activate PI3K. The PP1β was considered the possible hub-link between FSH and IGF1R signaling in granulosa cells (Law et al., 2015).

According to the mechanism described above, the requirement that PKA and PP1 facilitate the tyrosine phosphorylation of IRS1 through IGF1R could form the basis for the ability of FSH to promote the PI3K/AKT activation, which induces the gene expression. In line with this, covalent inhibition of IGF1R prevented FSH from phosphorylating downstream proteins in porcine SCs (48). Hence, IGF2 (by binding the IGF1R) and FSH could synergistically trigger the same molecular pathway.

### 4.3 IGF2 downregulated the expression of mitogens and FSHR

Incubation with rhIGF2 downregulated the expression of *GDNF*, *FGF2*, *SCF*, and *FSHR* genes. Among mitogens, this effect was particularly evident for GDNF whose lower secretion was significant at each concentration of IGF2. The secretion of GDNF and FGF2 by SCs is FSH-dependent (27). Both stimulate the proliferation of gonocytes (43, 79). GDNF also improves migration to the basement membrane (80) and the transition from an undifferentiated to a differentiated spermatogonial state (26). In contrast, SCF is involved only in the differentiation of spermatogonia (46). The effects of rhIGF2 incubation on gene expression were reversed in SCs pretreated with NVP-AEW541, which is an IGF1R inhibitor. The downregulation of mitogen gene expression represents a very interesting finding as it suggests the existence of a paracrine mechanism of regulation of spermatogenesis by which IGF2 released from GCs can inhibit the production of GDNF, FGF2, and SCF in SCs and downregulate FSHR, which makes SCs less sensitive to FSH, thus further contributing to the reduction of mitogen secretion. In other words, this is consistent with the presence of negative feedback that intervenes in the regulation of spermatogenesis. When the number of spermatozoa is high, the levels of IGF2 reaching SCs is greater, and, in turn, this would reduce the production of mitogens (especially GDNF), thus slowing the proliferation of gonocytes, their migration to the basement membrane, and their differentiation, thus keeping the number of spermatozoa in a definite range. Evaluation of the effect of rhIGF2 on the secretion of GDNF, FGF2, and SCF proteins by SCs is necessary to confirm this hypothesis. In this regard, we found that FSHR gene and protein expression goes in the same direction after incubation with rhIGF2, which highlights the decreased FSH sensitivity on SCs caused by rhIGF2.

Our results could be influenced by the absence of FSH synergistic activity. In fact, the lack of FSH in cultured SCs could cause a reduction in PKA activity, which may lead to decreased dephosphorylation of the inhibitory Ser/Thr residues on IRS1, causing a decreased activation of the PI3k/AKT pathway. This topic has not been investigated in this study; therefore, the effects of FSH on gene expression in SCs incubated with IGF2 deserve further investigation.

According to the data obtained, we hypothesize that rhIGF2 can induce downregulation of mitogens gene and FSHR gene and protein expression in SCs. Since we have demonstrated the presence of the IGF2 protein in spermatozoa, this could suggest the presence of negative feedback on the regulation of spermatogenesis. To the best of our knowledge, this is the first study showing this effect, so more studies are needed to confirm our findings.

### 4.4 IGF2 enhances the secretion of AMH and inhibin B by SCs

The evidence suggests that AMH and inhibin B could be used as markers of health, number, and maturation degree of SCs (81).

AMH is a dimeric glycoprotein that belongs to the transforming growth factor-β (TGF-β) superfamily, such as inhibin B, activins, and others (82). It is secreted by SCs and its main function is to reduce the regression of the Müllerian ducts, precursors of the female reproductive tract. Before puberty, FSH stimulates immature SCs to produce AMH (82). When puberty begins, the increase of intratesticular testosterone levels inhibits the transcriptional activation of AMH, thus its serum levels decrease (83–85). According to this, serum AMH levels reflect the number and/or degree of SC maturation.

Inhibin B is a heterodimeric glycoprotein, which plays a role in the negative feedback control of FSH secretion in men (86). At the beginning of puberty, the immature SCs switch to a mature and quiescent state and secrete higher amounts of inhibin B, which reflects the degree of FSH stimulation (87). An increase of serum inhibin B levels in post-pubertal SCs indicates an adequate exposure of these cells to FSH and intratubular testosterone.

The inhibin B transcription is stimulated by FSH, as mentioned previously, which acts through the cAMP-activated protein kinase A signaling pathway (88, 89), while the AMH transcription involves a nonclassical cAMP-response pathway requiring nuclear factor-κB (NKκB) and activating protein 2 (AP2) binding sites (90).

Our results show a downregulation of AMH and inhibin B transcription and an increased protein secretion after incubation with rhIGF2 at each concentration used. These effects were reversed by pre-treatment with NVP-AEW541. This suggests that rhIGF2 can modulate SC hormonal secretion by interacting with IGF1R. As previously reported, AMH levels are approximately ten to twenty times higher in childhood than in adulthood (85). When puberty begins, AMH levels initiate to decline in the bloodstream (85). Since AMH production is negatively regulated by intratubular testosterone concentrations, this decline is classically attributed to increased levels of this Leydig cell-derived hormone during the peripubertal phase (81). However, considering the negative effect of IGF2 on AMH secretion reported in the present study and the FSH-mediated expansion of the GC compartment, which accounts for most of the testes since puberty (81), a role of the GC-derived IGF2 in the downregulation of AMH in pubertal age and adulthood cannot be excluded.

GC-derived IGF2 may be part of a physiological paracrine regulatory mechanism between GCs, SCs, and Leydig cells, the dysfunction of which can lead to impaired spermatogenesis. For example, activin A is produced by Leydig cells during fetal life and exerts a proliferating effect on fetal SCs (91). Furthermore, there is also evidence for the production of retinol-binding protein 4 from spermatogonia in the developing testis, which in turn seems able to facilitate the transport of retinoic acid into SCs, thereby promoting the release of factors from SCs that regulate spermatogenesis (92). The ultrastructural features of SCs and the release of mitogens from these cells (e.g. GDNF, SCF) differ between patients with abnormal spermatogenesis (non-obstructive azoospermia) and those with preserved spermatogenesis (obstructive azoospermia). In particular, as reported in a comparative study on human testicular biopsies, SCs have smaller size, more vacuoles, deficient endoplasmic reticulum, and small and spindle-shaped nuclei, as well as a low expression of GDNF and SCF when spermatogenesis is altered (93). Another study reported lower levels of AMH in patients with non-obstructive azoospermia than in those with obstructive azoospermia (94), suggesting the presence of SC dysfunction in case of disrupted spermatogenesis. Whether SC dysfunction may arise as a primary disorder leading to abnormal spermatogenesis or, on the other hand, SC dysfunction may result from altered paracrine signaling of the dysfunctional GCs is a matter of debate.

### 4.5 IGF2 inhibits Sertoli cell proliferation

Although IGF2 is a mitotic factor, we found an inhibitory effect of IGF2 on SC proliferation, at the highest concentration used in our experimental model. This effect was not reversed by pre-incubation with the IGF1R non-competitive inhibitor NVP-AEW541, suggesting that it occurs independently of the IGF1R. This result may further support the existence of negative feedback on spermatogenesis regulation, as outlined above. This finding could, however, be explained by the presence of unknown interfering mechanisms or the absence of stimulating factors in this experimental model, such as FSH.

### 4.6 Limits of the study

Our results need to be taken with care, as the present experimental model does not resemble the complexity of testicular tissue. In fact, being an *in vitro* study carried out in porcine SCs, it cannot be excluded that the paracrine crosstalk with Leydig cells may affect the responsiveness of SCs to IGF2 *in vivo*. This limitation should be considered in further experimental studies.

## 5 Conclusions

The results of this study suggest that the IGF2 protein is present in human spermatozoa in varying amounts. To better understand its physiological function, we incubated porcine SCs with rhIGF2 and found that it downregulates the expression of mitogens, FSHR, and SC proliferation. This suggests the presence of negative feedback on the regulation of spermatogenesis, which allows the fluctuation of the number of spermatozoa within a certain range. We also found that IGF2 can increase the secretion of AMH and inhibin B, which are markers of SCs and tubular function. Porcine SCs have a high degree of similarity with human ones. Therefore, we speculate that similar mechanisms may also exist in human SCs. Hence, these findings may shed light on new diagnostic and therapeutic targets in the field of male infertility.

## Data availability statement

The original contributions presented in the study are included in the article/**Supplementary Material**. Further inquiries can be directed to the corresponding author.

## Ethics statement

The studies involving human participants were reviewed and approved by Institutional Ethic Committee of the University of Perugia (Ministry of Health authorization n. 971/2015-PR, 9/14/2015). Written informed consent for participation was not required for this study in accordance with the national legislation and the institutional requirements. The animal study was reviewed and approved by Institutional Ethic Committee of the University of Perugia (Ministry of Health authorization n. 971/2015-PR, 9/14/2015).

## Author contributions

RCa conceived the study, drafted the experiments, recruited the patients, wrote the manuscript; FM, MG and IA helped in drafting the experiments, performed them, and reviewed the manuscript; FM worked on the immunofluorescence and analyzed the results; CL performed the western blot; CB performed the real-time PCR; RCu helped in writing the first draft of the manuscript. MA carried out the ELISA dosages. SLV, RAC, and GL revised the manuscript; AEC managed the project, helped in drafting the experiments, supervised the project, and reviewed the final draft. All authors contributed to the article and approved the submitted version.

## Funding

This project was partially funded by the "ESHRE Travelling Fellowship 2020".

## Conflict of interest

The authors declare that the research was conducted in the absence of any commercial or financial relationships that could be construed as a potential conflict of interest.

## Publisher’s note

All claims expressed in this article are solely those of the authors and do not necessarily represent those of their affiliated organizations, or those of the publisher, the editors and the reviewers. Any product that may be evaluated in this article, or claim that may be made by its manufacturer, is not guaranteed or endorsed by the publisher.

## Supplementary material

Supplementary Figure 1 FSHR protein expression in HT29 cells. The immunofluorescence analysis of the FSHR protein expression in HT29 cell lines, used as negative control, shows no expression. Blu: DAPI (nuclei), Green: dy-light 488. Magnification 100x.

Supplementary Figure 2 Dot plots of FSHR-positive porcine SCs. SCs **(A)**. SCs + NVP-AEW541 **(B)**. SCs + IGF2 0.3 ng/ml **(C)**. SCs + IGF2 0.3 ng/ml + NVP-AEW541 **(D)**. SCs + IGF2 + 3 ng/ml **(E)**. SCs + IGF2 + 3 ng/ml + NVP-AEW541 **(F)**. SCs + IGF2 10 ng/ml **(G)**. SCs + IGF2 10 ng/ml + NVP-AEW541 **(H)**. Each dot plot is representative of 3 independent experiments. In each box, the histogram on the left is the same sample without the antibody for FSHR

Supplementary Figure 3 Histogram of CFSE-positive porcine SCs quantified by flow cytometry. SCs **(A)**. SCs + NVP-AEW541 **(B)**. SCs + IGF2 0.3 ng/ml **(C)**. SCs + IGF2 0.3 ng/ml + NVP-AEW541 **(D)**. SCs + IGF2 + 3 ng/ml **(E)**. SCs + IGF2 + 3 ng/ml + NVP-AEW541 **(F)**. SCs + IGF2 10 ng/ml **(G)**. SCs + IGF2 10 ng/ml + NVP-AEW541 **(H)**. Each histogram is representative of 3 independent experiments. In each box, the histogram on the left is the same sample unstained for CFSE.
