# Supplementary figures and images for: Sperm-carried IGF2 downregulated the expression of mitogens produced by Sertoli cells: A paracrine mechanism for regulating spermatogenesis?
Source: Front Endocrinol (Lausanne). 2022 Nov 29;13:1010796. doi: 10.3389/fendo.2022.1010796 (PMC9744929; doi:10.3389/fendo.2022.1010796)

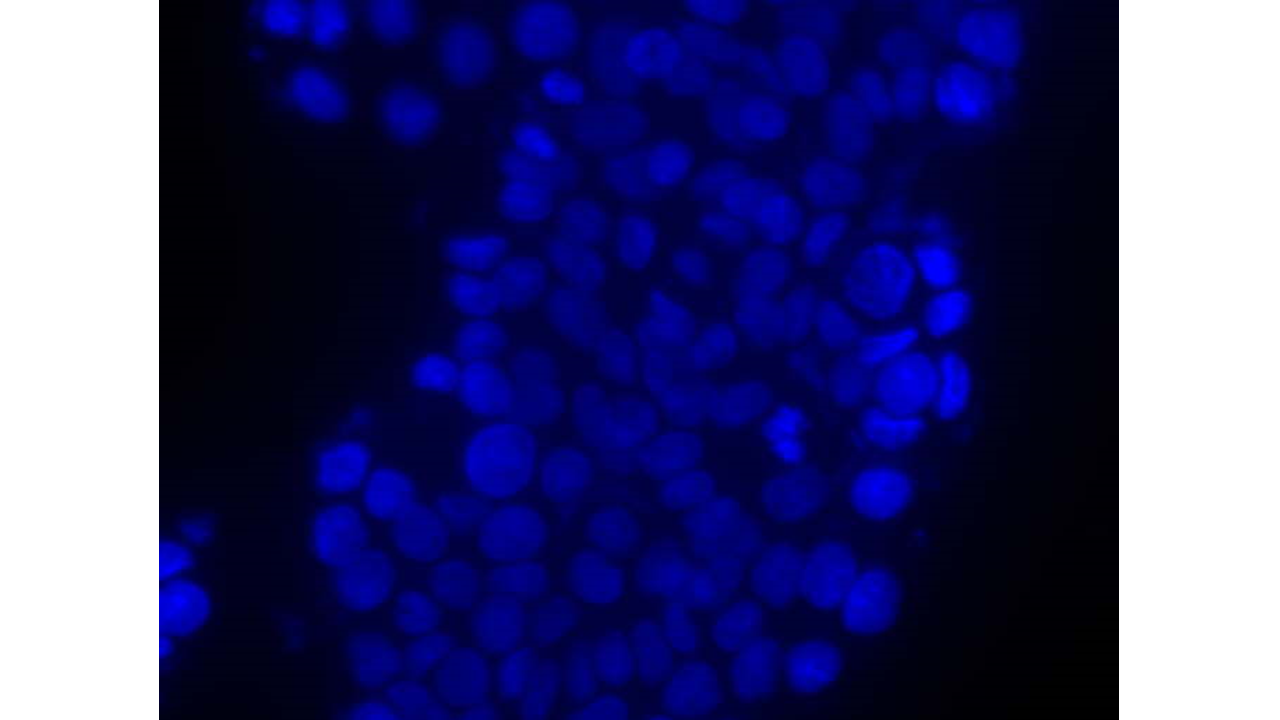

Supplement: Supplementary Figure 1 — FSHR protein expression in HT29 cells. The immunofluorescence analysis of the FSHR protein expression in HT29 cell lines, used as negative control, shows no expression. Blu: DAPI (nuclei), Green: dy-light 488. Magnification 100x. [file Image_1.tif]

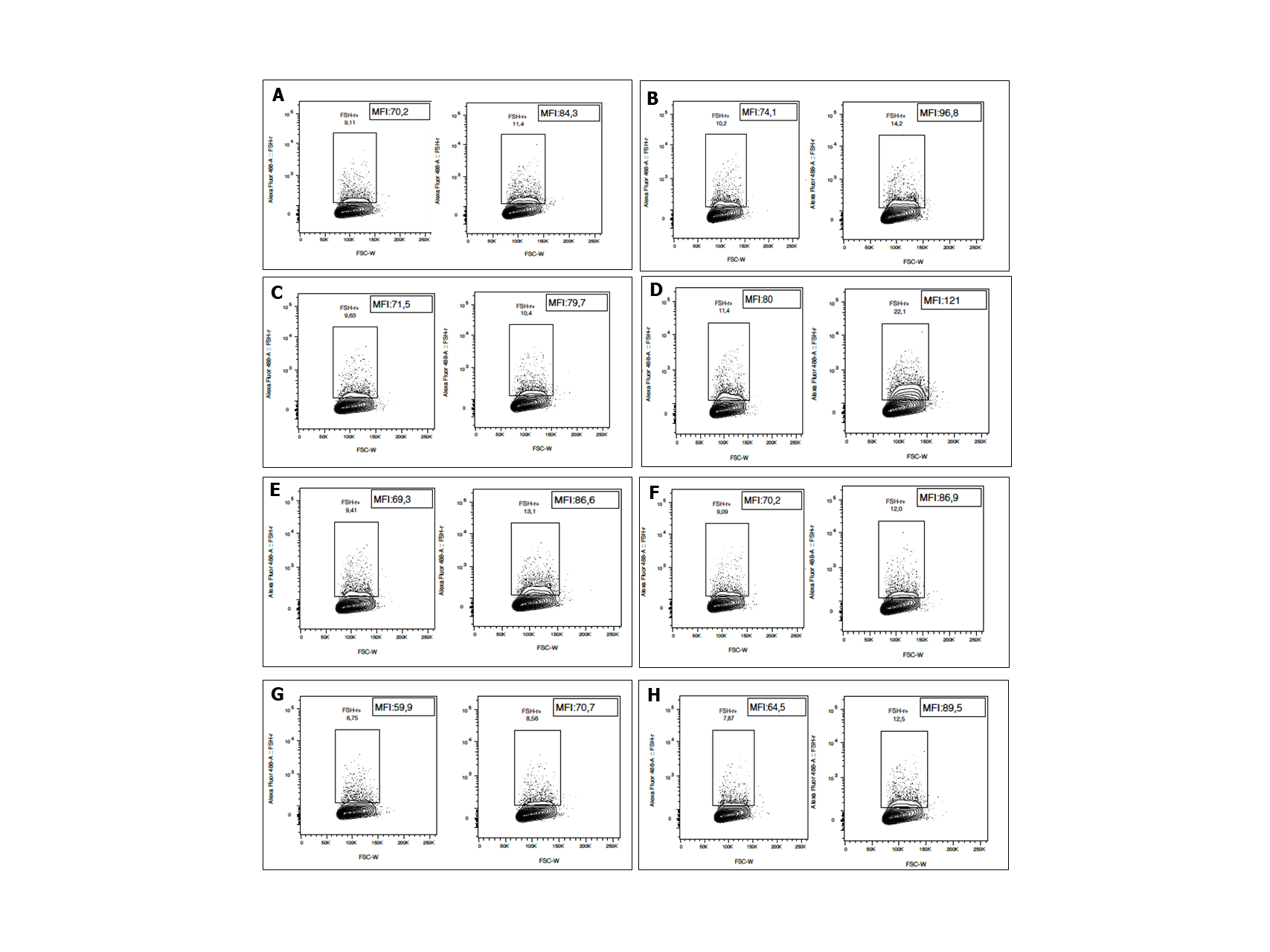

Supplement: Supplementary Figure 2 — Dot plots of FSHR-positive porcine SCs. SCs (A). SCs + NVP-AEW541 (B). SCs + IGF2 0.3 ng/ml (C). SCs + IGF2 0.3 ng/ml + NVP-AEW541 (D). SCs + IGF2 + 3 ng/ml (E). SCs + IGF2 + 3 ng/ml + NVP-AEW541 (F). SCs + IGF2 10 ng/ml (G). SCs + IGF2 10 ng/ml + NVP-AEW541 (H). Each dot plot is representative of 3 independent experiments. In each box, the histogram on the left is the same sample without the antibody for FSHR [file Image_2.tif]

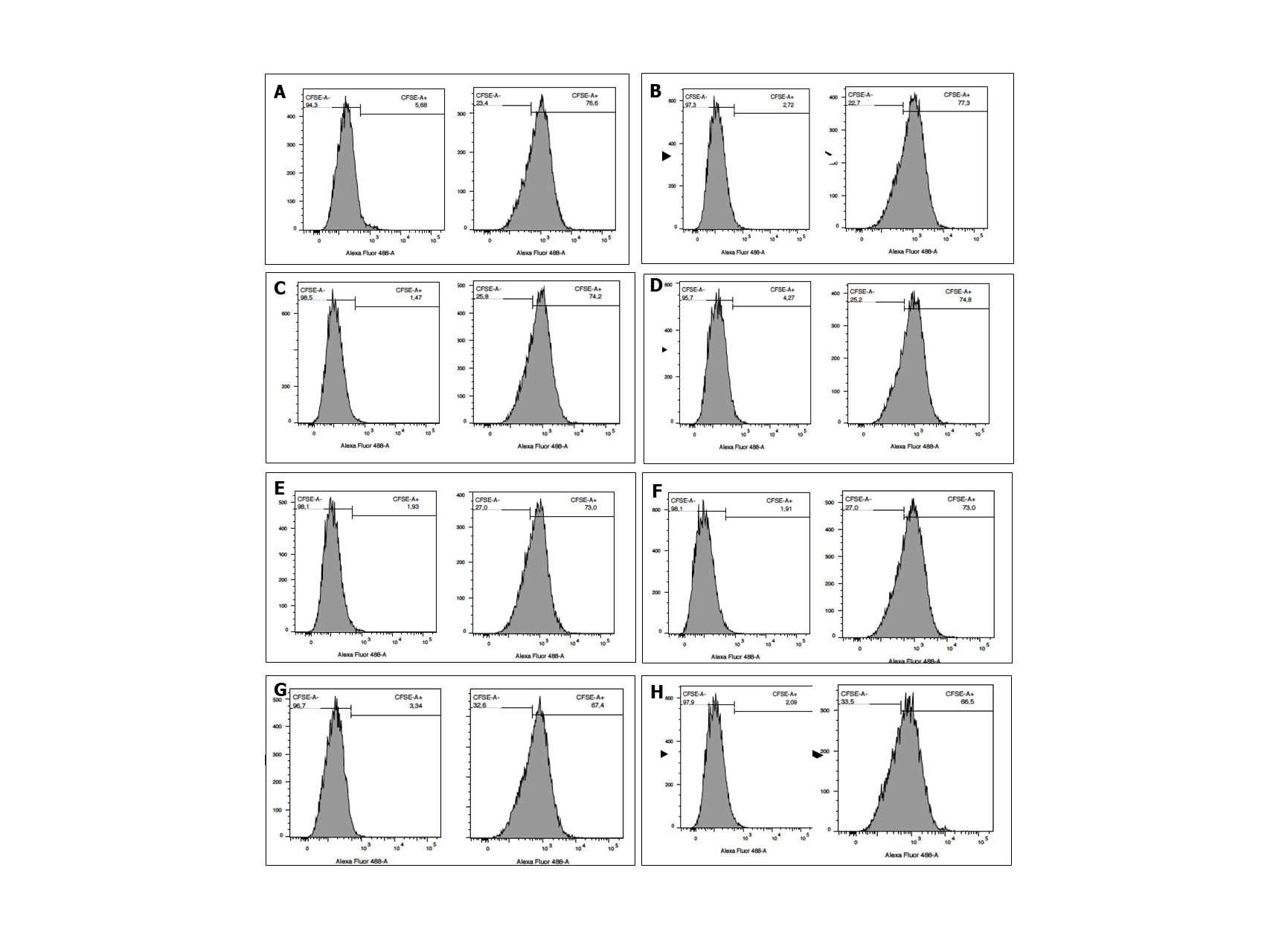

Supplement: Supplementary Figure 3 — Histogram of CFSE-positive porcine SCs quantified by flow cytometry. SCs (A). SCs + NVP-AEW541 (B). SCs + IGF2 0.3 ng/ml (C). SCs + IGF2 0.3 ng/ml + NVP-AEW541 (D). SCs + IGF2 + 3 ng/ml (E). SCs + IGF2 + 3 ng/ml + NVP-AEW541 (F). SCs + IGF2 10 ng/ml (G). SCs + IGF2 10 ng/ml + NVP-AEW541 (H). Each histogram is representative of 3 independent experiments. In each box, the histogram on the left is the same sample unstained for CFSE. [file Image_3.tif]
